# Baduanjin as an innovative intervention for mood disorders: evaluating efficacy and mechanisms

**DOI:** 10.3389/fpsyt.2025.1647619

**Published:** 2025-09-24

**Authors:** Jiping Chen, Yanyu Lu

**Affiliations:** ^1^ School of Physical Education, Shandong University, Jinan, China; ^2^ School of Innovation Design, Guangzhou Academy of Fine Arts, Guangzhou, Guangdong, China

**Keywords:** exercise, mood, Baduanjin, intervetion, mechanism

## 1 Introduction

Baduanjin is a traditional Chinese exercise that integrates physical movement, breathing techniques, and mental focus for overall wellness (1). It originated during the Song Dynasty (10th to 13th centuries AD) and is a form of Qigong, which is a broader practice aimed at cultivating “Qi” (energy) through exercises designed to promote health (2). Baduanjin consists of eight limb movements that focus on both body movement and breath control (3). In ancient times, millions of Chinese people practiced Baduanjin to improve and maintain their health. Recently, this exercise has gained worldwide popularity because of its many health benefits, ease of learning, and time efficiency (4). Studies have shown that joining a Baduanjin program can provide participants with various health benefits, such as a lower disease burden (5), improved sleep quality (6), and enhanced cognitive function (7). There is, however, limited information regarding the effects of Baduanjin on mood disorders.

The objectives of this opinion article are: 1 to gather clinical studies on Baduanjin for treating mood disorders, identify any evidence gaps, and guide future research; and 2) to summarize the potential mechanisms through which interventions may treat mood disorders based on existing research.

## 2 Potential of Baduanjin intervention in mood disorders—results from clinical studies

Current research suggests that Baduaniin positively affects individuals with mood disorders. To better understand its effectiveness and applicability across different types of mood disorders, this article will analyze patient responses from three key perspectives: physiology, psychology, and lifestyle habits.

Many studies have explored the effects of Baduanjin on individuals with mood disorders related to various underlying conditions. For instance, Wang et al. examined the impact of Baduanjin combined with five-element music therapy on 96 asymptomatic patients suffering from anxiety and depression due to COVID-19 (8). The results showed a significant reduction in scores on the 7-item Generalized Anxiety Disorder Scale (GAD-7) from baseline following treatment (Mean difference = 2.7, 95% CI = 2.3, 3.2, p < 0.001). This clinical trial sought to assess the feasibility and safety of Baduanjin in treating patients experiencing low mood due to COVID-19 infection during isolation. However, the study lacked blinding, meaning participants were aware of their assigned treatment group (treatment or control). This design flaw may have introduced a psychological expectancy effect, potentially influenced participants’ responses and skewed self-reported outcomes, such as anxiety and sleep quality. Future studies should consider employing a blinded design to mitigate bias and ensure more reliable results. In a follow-up study, Zhang and colleagues looked at how Baduanjin affects blood flow in the brain, risk factors for heart disease, and related mental health in 85 older adults at risk of having a stroke (9). The study found that older adults who practiced Baduanjin for one hour, five times a week for 12 weeks showed significant improvements in their mental health. They reported better mood, increased self-confidence, higher self-esteem, and an improved quality of life, both right after the program and during a follow-up 12 weeks later, compared to those who did not practice Baduanjin. This study is the first to show that Baduanjin, a traditional Chinese exercise, can help improve mental well-being in older adults at risk for stroke. However, the study did not use a blinded randomized controlled trial, which could have affected the results. Even if the effect size is estimated in the research report, small samples may not be representative of the entire high-risk population, limiting the universality of the research results.

Liu et al. examined the effects of Baduanjin exercise combined with rational emotive behavioral therapy (REBT) compared to escitalopram oxalate treatment paired with REBT on sleep and mood in stroke patients suffering from depression (10). At week 4, the Baduanjin group showed significantly better results in sleep latency, arousal index, Hamilton Anxiety Scale scores, Hamilton Depression Scale scores, and IL-6 levels compared to the control group (P < 0.05). After 8 weeks of treatment, the Baduanjin group demonstrated further improvements in these measures compared to the control group (P < 0.05), while both groups experienced significant enhancements from their baseline measurements (P < 0.001). These findings suggest that Baduanjin, when combined with cognitive-behavioral therapy, may provide greater psychological benefits than antidepressant medication, highlighting its potential role in improving mental health. The study primarily used the Hamilton Anxiety Rating Scale (HAMA) and the Hamilton Depression Rating Scale (HAMD) to assess emotional changes. Although these tools are widely used in clinical settings, they rely on subjective scoring and may be influenced by assessor bias. In the future, diversified assessment tools, such as a combination of physiological indicators and psychological assessments, could be introduced to enhance the reliability and comprehensiveness of the results.

From a psychological well-being perspective, two studies investigated the effects of the Eight-Section Exercise on the mental state of healthy university students. A study conducted by Chen et al. in 2016 assessed the impact of Baduanjin on mood states and executive function in 21 college students (11). The study found that an 8-week Ba Duan Jin intervention significantly reduced total mood disorder (TMD) scores when compared to a control group that participated in traditional relaxation training. Paired t-tests were conducted to analyze pre- and post-training mood states within each group. The results indicated a significant reduction in TMD scores for the Ba Duan Jin group [t = 6.45, p < 0.001]. In contrast, the control group showed no significant changes [t = −0.47, p = 0.64]. This research was among the first to explore the effects of Ba Duan Jin on mood disorders, yielding positive and statistically significant findings. However, the study had some limitations, including a small sample size and potential performance bias. The former may lead to insufficient statistical power of the research results, affecting the reliability and generalizability of the results. The latter may lead to experimenter bias or participant expectation effects influencing the experimental results. Li et al. investigated the effects of 12 weeks of Baduanjin on symptom intensity, stress, self-esteem, and mood in 101 college students, comparing them to those of regular physical activity (1). Both Baduanjin and regular exercise improved psychological outcomes like stress, self-efficacy, and mood, with no significant differences between the groups. These results suggest that Baduanjin has a similar effect on psychological well-being to traditional sports. The study employed a randomized, parallel-controlled design with a large sample. However, the control group in this study was only required to maintain their usual lifestyle and did not receive any intervention. This design may not be sufficient to clearly distinguish the effects of Baduanjin exercise from other potential factors (such as natural changes in lifestyle). If a more active control intervention (e.g., other forms of exercise or behavioral change interventions) could be added, it would help to better assess the unique effects of Baduanjin exercise.

In terms of lifestyle habits, Li et al. employed electroencephalography (EEG) and psychometric tools to systematically investigate differences in brain emotion regulation and the mechanisms underlying skill mastery in long-term versus short-term Baduanjin (12). The results revealed that engaging in long-term Baduanjin practice allowed participants to develop more effective cognitive reappraisal strategies, which significantly enhanced their ability to regulate emotions. This study was cross-sectional in design and could only reflect differences between groups at a given point in time. It did not examine the long-term effects of Baduanjin training on emotional regulation abilities. Although this type of design can reveal differences in training duration at a specific point in time, it cannot prove causality. From a neuroscientific standpoint, Baduanjin exercise promotes left frontal alpha lateralization, suggesting changes in the brain’s neuroplasticity associated with mood disorders. These findings deepen our understanding of how the prolonged practice of Baduanjin influences emotional management within the brain. Ultimately, this research may provide a solid theoretical foundation for promoting and popularizing the Baduanjin exercise.

## 3 Mechanisms of action of Baduanjin on mood disorders

Chemokines are a vital class of molecules in the immune system and have been thoroughly reviewed for their role in the development of mood disorders (13, 14). Various chemokines in the blood, including CCL2, CCL3, CCL4, CCL11, CXCL4, CXCL7, and CXCL8, have been shown to distinguish between patients with depression and those without (15). In particular, levels of CCL2, CXCL4, CXCL7, and CXCL8 were significantly higher in the blood of non-depressed patients compared to those with depression, while CCL4 showed the opposite trend (15). A recent clinical study by An et al. observed an increase in the expression of two chemokines, CXCL8 and CXCR4, in depressed patients after a 12-week Baduanjin intervention (16). CXCL8 is linked to neuroendocrine regulation and dysfunction of the hypothalamic-pituitary-adrenal axis (17, 18), whereas CXCR4 has been repeatedly identified as playing a role in neurogenesis (17, 19). Based on these findings, we hypothesize that Baduanjin may help regulate the function of the HPA axis and promote neurogenesis by upregulating the expression of these key chemokines, thus alleviating depressive symptoms.

Patients with depression often show deficits in the dopaminergic system, which may stem from dysfunction within affective frontal-limbic circuits. These circuits include the prefrontal cortex and various limbic structures, such as the hippocampus, amygdala, and anterior cingulate (20). NR4A2, a nuclear receptor and transcription factor with unique physiological properties, is recognized as a crucial regulator of the differentiation, survival, and maintenance of dopaminergic neurons (21). Initially, the NR4A2 gene polymorphism was suggested as a potential predictor of how effective antidepressants would be (22). Further research indicated a significant increase in NR4A2 expression in patients who underwent treatment with Baduanjin (FD = 3.448, P < 0.05). This suggests that the antidepressant effects of Baduanjin may be linked to the upregulation of NR4A2 expression, which supports the development of dopaminergic neurons in the midbrain and helps restore function within the frontal-limbic circuits (16).

A clinical study found that practicing Baduanjin for 12 weeks significantly impacted resting-state functional connectivity (rsFC) in the dorsolateral prefrontal cortex (DLPFC), a key area within the cognitive control network responsible for attention (23). The study also revealed that Baduanjin practice influenced connections in the insula, a brain region crucial for cognitive functions such as attentional reorientation triggered by stimuli, self-monitoring, and emotional awareness (23). These findings suggest that Baduanjin may help alleviate mood disorders by modulating the functions of cognitive control networks and enhancing cognitive performance. Additionally, previous research indicates that depression can inhibit the activity of the IL-17 pathway (24, 25). Research findings indicate a significant enhancement in IL-17 pathway activity following the Baduanjin in individuals diagnosed with depression. This suggests that Baduanjin may exert its antidepressant effects by upregulating the IL-17 pathway (16).

## 4 Discussion

**Figure 1:**
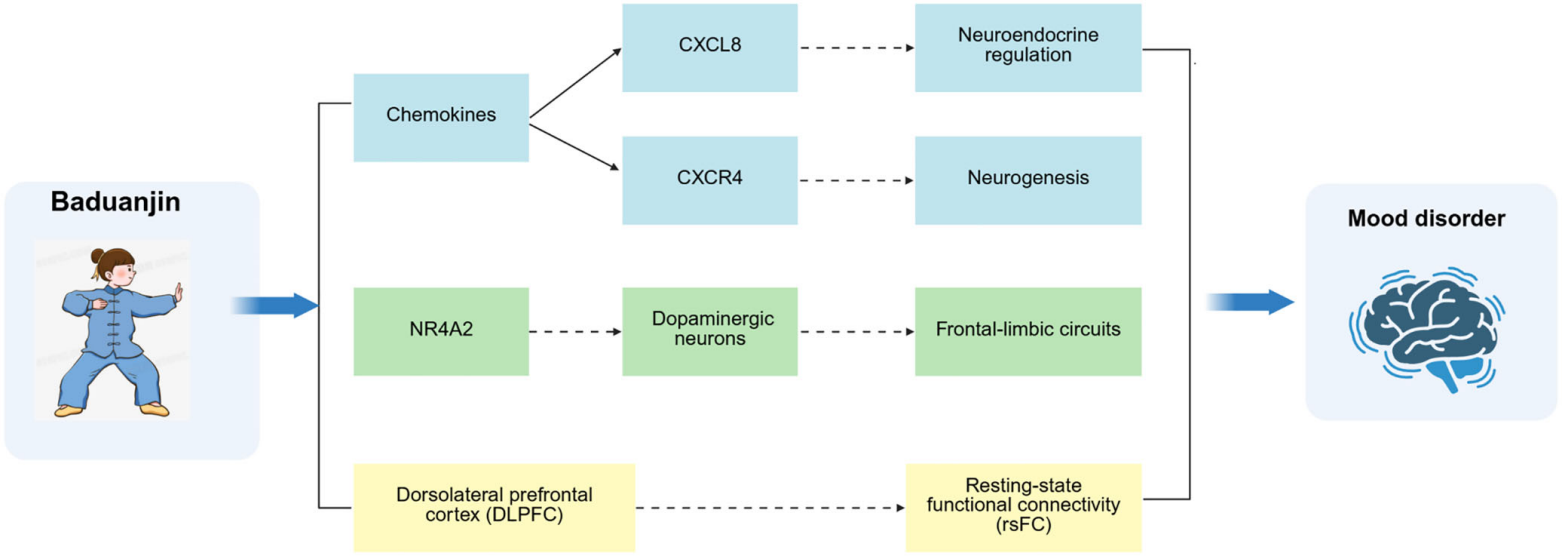
The mechanisms of action of Baduanjin on mood disorders.

Current evidence suggests that Baduanjin treating mood disorders by upregulating chemokines (CXCR4, CXCL8), NR4A2 expression, the IL-17 pathway, and modulating cognitive (attentional) control networks (**Table 1**, **Figure 1**). However, the current study is subject to several limitations. Firstly, as a non-pharmacological intervention, there are issues with the methodology of the research. Specifically, many studies have small sample sizes, which may affect the statistical significance and generalizability of the results. In addition, many studies did not use a blinded design, which may have led to the experimenter or participant’s expectations influencing the results, thereby reducing the objectivity of the research. More importantly, many studies lacked effective control groups, making it difficult to confirm causality and potentially overestimating the effectiveness of the treatment. Secondly, this study analyzed clinical data based on a Chinese population, which makes the results of the study may not be fully applicable to populations from other regions or different ethnic groups. Although the Chinese population is representative of the characteristics of certain diseases and health problems, factors such as genetics, environment, culture and lifestyle in different regions may lead to differences in study results. Therefore, the results of studies limited to a single population may not be widely generalizable. Lastly, prior research predominantly emphasizes short-term interventions and follow-up assessments, thereby complicating the evaluation of the long-term effects of Baduanjin on mood disorders.

**Table 1:** Characteristics of included studies.

Study	Participant information	Content of the intervention	Primary outcome measures	Statistical tests	Major performance metrics
Chen et al. (11)	Baduanjin group: 21 undergraduate or graduate students; Control group: 21 undergraduate or graduate students; All: 61.9% of females; mean (SD) age = 22.5 ± 2.0.	Baduanjin group: 90 minutes per session with five sessions per week for 8 weeks. Control group: 8 weeks progressive muscle relaxation training.	(1) the shortened Profile of Mood States (POMS); (2) total mood disturbance (TMD) scores.	(1) the paired t-tests; (2) the repeated measures ANOVA; (3) the analysis of Variance (ANOVA).	(1) the Baduanjin group showed a significant reduction in TMD score after the training, indicating a marked improvement in mood (p < 0.001); (2) the control group did not show significant changes in their mood (p = 0.64).
Li et al. (1)	Baduanjin group: 101university students; 85.1% of females; mean (SD) age = 20.63 ± 1.03. Control group: 105 university students; 80% of females; mean (SD) age = 20.92 ± 1.15.	Baduanjin group: 1-hour per session with five sessions per week for 12 weeks. Control group: without any intervention.	(1) self-reported.	(1) T-test or Mann-Whitney test; (2) linear regression model; (3) logistic regression model; (4) intention-to-treat (ITT) analysis.	No significant change in self-reported psychological symptom intensity, stress, self-efficacy was observed at the end of 12-week intervention period.
Li et al. (12)	Long-term Baduanjin learning group: 22 university students; 63.6% of females; mean (SD) age = 19.68 ± 1.13. Short-term Baduanjin learning group: 21 university students; 42.9% of females; mean (SD) age = 19.90 ± 0.70.	12-minute set of Baduanjin	(1) the Emotion Regulation Questionnaire (ERQ) (2) the Profile of Mood States-Short Form (POMS-SF).	(1) independent samples t-tests; (2) Pearson correlation analyses; (3) repeated measures analysis of variance (ANOVA); (4) paired-sample t-tests.	(1) ERQ: long-term group: Higher cognitive reappraisal scores (33.68 ± 5.16) compared to the short-term group (29.62 ± 4.86), with a significant difference (p = 0.01, Cohen’s d = 0.81); (2) POMS-SF: no significant differences were found between the groups for most mood states (e.g., tension, anger, fatigue, depression, vigor, confusion, esteem-related affect).
Liu et al. (10)	Baduanjin group: 50 patients with stroke; 38% of females; mean (SD) age = 58.86 ± 10.83. Control group: 50 patients with stroke; 42% of females; mean (SD) age = 56.22 ± 11.54.	Baduanjin group: 30 minutes per session with twice a day or 8 weeks. Control group: usual care.	(1) the Hamilton Depression Scale (HAMD); (2) the Hamilton Anxiety Scale (HAMA).	(1) the independent sample t-test; (2) the paired t-test; (3) the Mann-Whitney U test; (4) Chi-square test; (5) multiple imputation.	(1) anxiety: the Baduanjin group showed a significant reduction in anxiety, with the HAMA score decreasing from 24.68 ± 7.38 to 11.60 ± 4.46 at 8 weeks (p < 0.001); (2) depression: the Baduanjin group showed a significant reduction in depression, with the HAMD score decreasing from 21.48 ± 8.03 to 8.54 ± 3.44 at 8 weeks (p < 0.001).
Wang et al. (8)	Baduanjin group: 96 asymptomatic patients with COVID-19 infection; 37.5% of females; age range 18–60. Control group: 81 asymptomatic patients with COVID-19 infection; 37% of females; age range 18–60.	Baduanjin group: 10–15 minutes per session with twice a day until health. Control group: usual care.	(1) the 7-item Generalized Anxiety Disorder scale (GAD-7); (2) the Patient Health Questionnaire (PHQ-9).	(1) one-way analysis of covariance (ANCOVA); (2) T-tests or Wilcoxon Rank Sum Test; (3) chi-square or Fisher’s Exact Test.	(1) the treatment group showed significant reductions in anxiety (GAD-7 score decreased by 2.7 points, p < 0.001) and depression (PHQ-9 score decreased by 3.7 points, p < 0.001), while the control group showed no significant changes; (2) between-group comparisons revealed significant differences in both anxiety (MD = 2.9, p < 0.001) and depression (MD = 3.6, p < 0.001).
Zheng et al. (9)	Baduanjin group: 85 adults with a high risk of ischemic stroke; 64.7% of females; mean (SD) age = 60.53 ± 6.29. Control group: 85 adults with a high risk of ischemic stroke; 63.5% of females; mean (SD) age = 59.75 ± 6.34.	Baduanjin group: 1-hour per session with five sessions per week for 12 weeks. Control group: without any intervention.	(1) the Brief Profile of Mood States (BPOMS); (2) the Rosenberg Self-Esteem Scale; (3) the Chinese version of the Social Functioning 36 (SF-36) scale; (4) the Pittsburgh Sleep Quality Index (PSQI).	(1) two-sample t-tests or Mann-Whitney tests; (2) linear Mixed Models (LMMs); (3) multiple Imputation Method.	(1) mood: significant improvement in mood scores at both post-intervention and follow-up stages; (2) self-confidence and self-esteem: enhanced self-confidence and self-esteem in the Baduanjin group; (3) quality of Life: significant improvements in the quality of life scores.

Future research should focus on extending both the intervention and follow-up periods to more comprehensively evaluate the long-term effects of Baduanjin on mood disorders and associated clinical outcomes. In order to further validate the results of this study and to improve its generalizability, future studies should be conducted in different populations, including but not limited to other Asian countries, European and American countries, and populations of different ethnic backgrounds. Genetic, lifestyle and environmental differences among different populations may have an important impact on the results of clinical studies. Therefore, cross-cultural and multinational multicenter clinical studies will contribute to a more comprehensive understanding of the manifestations and treatment effects of related diseases. Moreover, increasing the sample size and refining the design of randomized controlled trials would be instrumental in confirming the sustained efficacy and elucidating the underlying mechanisms of Baduanjin. Additionally, enhancing blinding procedures and diversifying the sample population are essential steps to ensure greater objectivity and broader applicability of the findings across diverse demographic groups.
